# Global Evolution of Microbiological Patterns and Pharmacological Guidance in the Management of Peritoneal Dialysis-Associated Peritonitis: An Evolution of Epidemiology and Clinical Practice

**DOI:** 10.3390/pathogens15101030

**Published:** 2026-09-30

**Authors:** Ola Suliman, Henry H. L. Wu, David Lewis, Philip A. Kalra, Rajkumar Chinnadurai

**Affiliations:** 1 Donal O’Donoghue Renal Research Centre, Salford Royal Hospital, Northern Care Alliance NHS Foundation Trust, Stott Lane, Salford M6 8HD, UK; hon.wu@sydney.edu.au (H.H.L.W.); david.lewis@nca.nhs.uk (D.L.); philip.kalra@nca.nhs.uk (P.A.K.); 2 Division of Cardiovascular Sciences, Faculty of Biology, Medicine and Health, The University of Manchester, Manchester M13 9PL, UK; 3 Department of Renal Medicine, Royal North Shore Hospital, Northern Sydney Local Health District, St. Leonards, NSW 2065, Australia

**Keywords:** peritoneal dialysis, peritoneal dialysis-associated peritonitis, antibiotics, microorganisms, global, clinical outcomes, inflammation

## Abstract

Peritoneal dialysis (PD)-associated peritonitis remains a major cause of treatment failure, hospitalisation, catheter loss and transition to haemodialysis despite reductions in incidence over the decades. This narrative review examines the global evolution of microbiological epidemiology and pharmacological management of PD-associated peritonitis, looking at global trends in microbiological epidemiology and exploring how treatment recommendations have evolved in response to changing pathogen distribution, antibiotic resistance patterns and associated clinical outcomes. Although Gram-positive organisms remain the predominant pathogens in most PD programmes, recent evidence demonstrates marked geographical and centre-level heterogeneity with rising clinical importance of Gram-negative, polymicrobial, fungal and culture-negative infections in certain regions. Antibiotic resistance has emerged as an additional challenge, including methicillin-resistant staphylococci, vancomycin-resistant enterococci, extended-spectrum β-lactamase-producing Enterobacterales and multidrug-resistant non-fermenting Gram-negative organisms. These changes challenge conventional empirical regimens and emphasise the importance of programme-specific microbiological surveillance. In parallel, pharmacological management has evolved considerably from relatively standardised empirical antibiotic combinations towards an individualised approach incorporating intraperitoneal administration, local susceptibility profiles, organism-directed therapy, pharmacokinetic optimisation, therapeutic drug monitoring and antifungal prophylaxis. However, substantial evidence gaps remain, particularly regarding antibiotic resistance patterns globally, optimal dosing in automated PD, intraperitoneal stability and clinical efficacy of newer antimicrobial agents.

## 1. Introduction

Peritoneal dialysis (PD)-associated peritonitis remains one of the most significant complications of PD and is a major contributor to PD loss, hospitalisation and mortality. According to the International Society for Peritoneal Dialysis (ISPD) 2016 guidelines, PD-associated peritonitis contributes to approximately 16% of PD-related deaths [1]. Encouragingly, the incidence of PD-associated peritonitis has declined over the past three decades due to improvements in patient education, PD connection systems, exit-site care, prophylactic antibiotics and continuous quality improvement programmes. A recent systematic review published in PDI (Peritoneal Dialysis International) in 2022 of registry data from 33 countries demonstrated a reduction in global peritonitis rates from approximately 0.60 episodes per patient-year to around 0.30 episodes per patient-year between 1990 and 2019; nevertheless, it continues to represent a major barrier to long-term PD success [2]. Globally, PD-associated peritonitis represents a major healthcare burden where treatment frequently requires prolonged antibiotic therapy, hospital admission, catheter replacement and temporary or permanent transition to haemodialysis, all of which substantially increase healthcare costs and reduce the long-term sustainability of PD programmes. The ISPD (International Society of Peritoneal Dialysis) 2022 guidelines recommend that centres achieve fewer than 0.4 episodes per patient-year and that 80% of patients remain peritonitis free per year, a benchmark that has been attained by high-performing programmes but remains unmet in many regions worldwide [3].

Over the years, there has been substantial evolution in the way we investigate and treat PD-associated peritonitis, as it is a complication that has shaped the success of PD. Alongside improvements in preventive strategies and clinical management, the microbiological epidemiology of PD-associated peritonitis has evolved considerably. Changes in organism distribution, antibiotic resistance and diagnostic practices have influenced both the management of peritonitis and subsequent updates to international guidelines. A large analysis from the Australia and New Zealand Dialysis and Transplant (ANZDATA) Registry reported that 53.4% of peritonitis episodes were caused by Gram-positive organisms, 23.6% by Gram-negative organisms, 11% were polymicrobial and 13% were culture-negative [4]. While Gram-positive organisms remain the predominant pathogens, an increasing proportion of Gram-negative infections has been reported, promoting changes in empirical antibiotic therapy and emphasising the need for ongoing surveillance [5].

Antibiotic resistance has emerged as one of the greatest challenges in the management of PD-associated peritonitis. Increasing prevalence of methicillin-resistant *Staphylococcus aureus* (MRSA), vancomycin-resistant enterococci (VRE), extended-spectrum β-lactamase (ESBL)-producing Gram-negative organisms and other multidrug-resistant pathogens has complicated empirical treatment strategies and adversely affected outcomes. Despite growing recognition of these resistance patterns, their global distribution and trends have not been comprehensively mapped in the literature.

Successive ISPD guidelines have adapted treatment recommendations in response to these microbiological changes. Current practice guidance emphasises prompt administration of intraperitoneal antibiotics, empirical coverage of both Gram-positive and Gram-negative organisms, antifungal prophylaxis during antibiotic therapy, and earlier catheter removal in fungal, refractory, relapsing, or non-resolving episodes [1,3].

Despite the above, the healthcare burden of PD-associated peritonitis remains large, with vast geographical variation persisting in both the incidence of peritonitis and the microbiology affecting clinical outcomes owing mainly to differences in healthcare infrastructure, catheter insertion practices, prophylactic and preventative practices, patient training and education, microbiological laboratory capacity, antibiotic prescribing, socioeconomic factors and local pathogen ecology, which contributes to regional variability in causative organisms and clinical outcomes.

Understanding the microbiology of PD-associated peritonitis along with its global evolution is core to solving the problem, consequently influencing targeted antibiotics and aiding in generating standardised guidelines.

## 2. Global Shifts & Trends in Microbiological Patterns

Trends of PD-associated peritonitis vary regionally and globally according to infrastructure, staffing and centre-level expertise. Despite the broad trends described above, recent evidence suggests that the microbiological evolution of PD- associated peritonitis has not followed a uniform global pattern. Improvements in peritonitis incidence and rates have therefore not occurred equally across all organism groups, regions and services.

The Peritoneal Dialysis Outcomes and Practice Patterns Study (PDOPPS) provided one of the clearest multinational demonstrations of this geographical variation [6]. Among 7051 patients treated in 209 facilities across Australia, New Zealand, Canada, Japan, Thailand, the United Kingdom and the United States, 2272 peritonitis episodes were recorded from 2014 to 2017. Peritonitis rates varied considerably between countries, from 0.26 episodes per patient-year in the United States to 0.38 episodes per patient-year in Thailand, while 10% of participating facilities reported rates exceeding 0.50 episodes per patient-year. Although the broad microbiological distribution was similar across most countries, Gram-negative and culture-negative episodes were more frequent in Thailand [6] (Table 1). This demonstrates that national estimates could obscure large variations between centres and reporting bias and that microbiological patterns are influenced by both local programme performance and geography.

The observed international variation should not be interpreted as a purely geographical phenomenon; instead, the multifactorial differences described earlier in patient education, initial PD training, staffing, catheter-care procedures, antibiotic prophylaxis, access to clean water and microbiological service-level may all affect the organism profile recorded by a programme. PDOPPS found that longer initial training and prophylactic antibiotic administration at catheter insertion were associated with lower peritonitis rates, explaining that some of the variation is potentially modifiable [6]. Over the next section, we will review studies from across various regions to examine trends globally.

### 2.1. Australia and New Zealand

Environmental and climatic conditions may nevertheless contribute to regional differences. Registry data from Australia demonstrated higher fungal and overall peritonitis rates in tropical areas compared with temperate or subtropical regions, together with a shorter time to the first peritonitis episode following the commencement of PD. Culture-negative peritonitis was significantly less likely in tropical regions [7]. A related multi-registry analysis of 6610 Australian PD patients found seasonal variation in overall peritonitis due to specific microorganisms, demonstrating autumn and summer peaks in fungal peritonitis/Gram negative organisms and summer peaks in *pseudomonas* infection, with Corynebacteria peaking in winter [8]. These observational data are biologically logical given the environmental reservoirs of these organisms and the potential effects of heat and humidity on bacterial proliferation, skin colonisation and exchange-related contamination. However, climatic associations have not been reproduced consistently enough in the literature to justify any seasonal alteration of empirical antibiotic regimens, and local surveillance of trends remains more informative than climate alone.

The importance of Gram-negative peritonitis has become particularly apparent because its clinical burden is often disproportionate to its frequency. Enteric organisms, such as *Escherichia coli* and *Klebsiella* species, may result from gastrointestinal translocation or intra-abdominal pathology, whereas *Pseudomonas*, *Acinetobacter* and other non-fermenting Gram-negative bacilli may also reflect environmental or water-associated exposure. These organisms cannot be considered a homogeneous group because their antibiotic susceptibility, propensity for biofilm formation, and clinical outcomes differ substantially.

In an Australian registry study of 191 PD peritonitis episodes, *Pseudomonas* accounted for 5.3% of all peritonitis episodes and was associated with high rates of catheter removal and permanent transfer to haemodialysis (HD). Treatment with two active anti-pseudomonal agents was associated with a lower risk of permanent haemodialysis transfer than single-agent therapy, while prompt catheter removal was associated with a lower risk of death than continued antibiotic treatment alone [9]. These findings consolidated *Pseudomonas* peritonitis as a high-risk clinical entity and serious complication of PD rather than simply another cause of Gram-negative infection.

Other Gram-negative organisms also carry important risks. In a separate ANZDATA analysis, these episodes were associated with increased hospitalisation, catheter removal and permanent HD transfer compared to other organisms, and the overall antibiotic cure rate was only 59% [10]. Nevertheless, species–specific outcomes vary. A multicentre registry analysis (ANZDATA) of 253 *Acinetobacter* episodes found that 74% of solitary infections were cured with antibiotics alone and that outcomes were more favourable than those observed with *Pseudomonas*, other Gram-negative, fungal or polymicrobial infections [11]. Conversely, in a separate region, a Hong Kong cohort identified multidrug-resistant *Acinetobacter* in 18% of episodes and carbapenem-resistant isolates in 8% of episodes, of which up to 80% of peritonitis episodes caused by carbapenem resistance resulted in catheter loss or mortality, demonstrating that favourable aggregate outcomes do not eliminate the risk caused by local resistance patterns [12].

The impact of resistance is illustrated by *Staphylococcus aureus* (*S. aureus*). In an Australian cohort of 503 episodes, *S. aureus* peritonitis was associated with higher relapse rates than non-*S. aureus* infection. *MRSA* was independently associated with an increased risk of permanent HD transfer, and adverse outcomes were more frequent when vancomycin was not used after MRSA had been identified [13]. Clinically important epidemiological transition is not limited to changes in the frequency of particular microorganisms but also includes the emergence of resistant subgroups within organisms that have long been recognised as causes of PD peritonitis.

Polymicrobial infection provides a further indication that broad organism categories may obscure clinically important phenotypes. In another ANZDATA registry analysis, polymicrobial infection accounted for 10% of episodes. Compared with single-organism peritonitis, it was associated with greater hospitalisation, catheter removal, permanent HD transfer and mortality. Outcomes were principally determined by the organisms recovered; episodes involving only Gram-positive bacteria frequently responded to medical treatment, whereas the presence of Gram-negative bacteria or fungi was associated with worse prognosis [14]. Polymicrobial infection involving enterococci has emerged as a topic of discussion. The frequency, predictors, treatment and clinical outcomes of enterococcal peritonitis were investigated in 4675 patients receiving PD in Australia; among 116 enterococcal episodes, 45% were polymicrobial, and the isolation of additional organisms was the principal predictor of adverse outcomes. Polymicrobial enterococcal infection was associated with catheter removal in 52% and permanent HD transfer in 50% of episodes whilst clinical outcomes were broadly comparable for pure enterococcal and non-enterococcal peritonitis [15]. This supports careful evaluation for gastrointestinal or other intra-abdominal pathology when enteric or multiple organisms are isolated.

Culture-negative peritonitis represents another major source of apparent geographical and temporal variation. Its frequency is influenced not only by prior antibiotic administration and low microbial burden but also by how PD effluent is collected, transported, inoculated and processed. An ANZDATA study of 435 culture-negative episodes found that previous antibiotic treatment was more frequent than in culture-positive peritonitis. Culture-negative episodes had favourable outcomes, including higher rates of cure with antibiotics alone and lower rates of catheter removal and permanent HD transfer [16]. However, this should not lead to diagnostic complacency, particularly when symptoms persist or episodes relapse.

Differences in microbiological methodology can also distort comparisons across time. The introduction of bedside inoculation into blood-culture bottles, automated incubation, improved species identification and molecular techniques may increase the detection of fastidious, anaerobic or atypical organisms that would previously have been classified as culture-negative.

Fungal and mycobacterial peritonitis remain uncommon but have a disproportionate effect on technique survival. Australian registry data demonstrated that fungal, mycobacterial and anaerobic infections were among the organisms most frequently requiring catheter removal [4]. Fungal peritonitis is strongly associated with previous bacterial peritonitis and preceding antibiotic exposure and frequently results in permanent cessation of PD [17]. These infections show why trends based solely on episode frequency can be misleading; rare organisms may contribute substantially to catheter loss, hospitalisation and mortality even when they represent only a small proportion of total episodes.

The observed global pattern in this region is therefore not a simple replacement of Gram-positive by Gram-negative infection. Gram-positive organisms remain central to PD-peritonitis epidemiology, but their relative dominance has been modified by the preferential reduction in preventable touch-contamination episodes, persistent regional disparities, changing resistance within established pathogens and improved recognition of atypical infections.

### 2.2. North America

Earlier large-scale North American data showed that Gram-positive organisms accounted for approximately 62% of peritonitis episodes in the United States and 61% in Canada, with Gram-positive organisms accounting for the majority of exit-site infections in the US (69%) and Canada (76%) [18]. In contrast, a longitudinal Canadian study comparing episodes occurring between 2005 and 2014 with the same centre’s earlier experience found that the overall microbiological distribution remained broadly similar over the decade but resistance patterns within both Gram-positive and Gram-negative isolates had changed despite rates being stable [19]. Stability in the proportion of Gram-positive and Gram-negative episodes can obscure substantial changes in methicillin resistance, cephalosporin susceptibility and the effectiveness of empirical regimens [19]. Canadian multicentre data have also shown that the microbiological pattern may change across successive episodes within the same patient, with recurrent peritonitis caused by the same organism being due to coagulase-negative Staph in 65.7% of the cases [20].

These observational studies suggest that the principal North American shift has not been a wholesale replacement of Gram-positive infection by other infections but rather a reduction in some contamination-related episodes alongside increasing clinical concern regarding resistant staphylococci. However, due to limited data available from the region compared to Australia/ New Zealand, it is challenging to accurately extrapolate practices and compare between regions.

### 2.3. Europe

The European experience similarly demonstrates that microbiological change is organism-specific. A London-wide audit where the outcome of peritonitis in PD patients attending 12 PD units in the Thames area between 2002 and 2003 confirmed that peritonitis remained a major cause of morbidity despite substantial historical reductions in overall incidence; coagulase-negative staphylococcus (CNS) was the most common cause, accounting for around 30% of all peritonitis episodes, including recurrences, followed by non-pseudomonas gram negatives and *Staphylococcus aureus*. Cure rates were 77.2% for CNS, 46.6% for *S. aureus*, and 7.7% for MRSA. The cure rate for *Pseudomonas* was 21.4% and 56.7% for other Gram-negative bacteria, with marked variation in rates and outcomes between PD units [21]. This centre-level variation indicates that microbiological epidemiology is strongly influenced by programme organisation and infection-control practices rather than national geography alone.

Long-term data from Spain provide important evidence against a simple worldwide Gram-negative shift. In a 30-year analysis of 1061 peritonitis episodes, the incidence of staphylococcal and Gram-negative peritonitis declined, whereas streptococcal and polymicrobial infections became proportionally more prominent. Streptococci were isolated in more than half of polymicrobial episodes, suggesting that prevention strategies effective against staphylococcal touch contamination may have had less impact on oral, gastrointestinal or mixed-source infections [22]. A separate Spanish analysis showed that enteric peritonitis is a heterogeneous group ranging from single-organism infections to polymicrobial episodes associated with more severe intra-abdominal disease and worse outcomes, especially if isolates in the polymicrobial episodes had intestinal anaerobes and/or *Enterococcus faecium* [23].

These findings show that even within Europe, the direction of microbiological change differs from that reported in some Asian or Australasian cohorts. The growing relative importance of streptococci and polymicrobial infection in Spain contrasts with reports of increasing Gram-negative or resistant staphylococcal disease elsewhere.

Culture-negative infection also varies considerably across European programmes. A UK survey involving 53 PD centres reported culture-negative proportions ranging from approximately 5% to 38%, accompanied by substantial variation in effluent collection, transport and laboratory-processing procedures [24]. This degree of variability shows that potential differences in organism distribution may reflect centre diagnostic performance rather than true epidemiological variation.

### 2.4. East and Southeast Asia

Asian studies demonstrate some of the clearest regional variation in Gram-negative infection and antibiotic resistance. Southern Taiwanese data have identified *Klebsiella pneumoniae* as an important cause of both monomicrobial and polymicrobial peritonitis, while a single-centre 10-year study has documented increasing methicillin resistance among staphylococcal isolates [25,26]. These findings suggest simultaneous changes in the frequency of enteric Gram-negative organisms and the susceptibility of established Gram-positive pathogens.

Chinese studies have similarly highlighted the clinical importance of resistant *Enterobacterales*. In a South Chinese cohort, *E. coli* was the leading cause of monomicrobial Gram-negative peritonitis (59.2%). ESBL-producing strains accounted for 35.5% of *E. coli* peritonitis. Previous episodes of peritonitis were associated with extended-spectrum β-lactamase-producing *Escherichia coli* (*ESBL*), and a Charlson Comorbidity Index (CCI) above 3 or diabetes mellitus or hypoalbuminemia (≤30 g/L) on admission were associated with poorer outcomes and treatment failure [27]. More recent Chinese data continue to identify changing Gram-negative susceptibility and multidrug resistance as important determinants of treatment failure. However, these observations remain programme-specific and may not apply uniformly across China or Asia.

Taiwanese experience has also highlighted the continuing relevance of tuberculous peritonitis in endemic areas where the reported annual TB incidence of the dialysis population (493.4/100,000) was 6.9 times higher than that of the general population (71.1/100,000) in Taiwan and where delayed diagnosis may result from an initially culture-negative or indolent presentation [28]. Over 50% of the patients diagnosed with tuberculous peritonitis had a white cell count (WCC) <100 cells/μL in PD fluid [28].

Thailand provides an important example of how laboratory infrastructure can shape reported microbiology. In a prospective study using paired specimens, culture negativity was approximately 38% when samples were processed by local laboratories but only 12% when corresponding specimens were processed by a central reference laboratory. Nurse-led sampling, rapid transport, centrifugation and greater microbiological capacity improved organism recovery [29]. The high culture-negative burden reported in some lower-resource settings must therefore be interpreted partly as a diagnostic-system limitation rather than evidence of a biologically distinct form of peritonitis.

### 2.5. Latin and South America

Latin American evidence is less extensive than from Australia, Europe or East Asia, but available studies demonstrate further regional diversity. Brazilian cohorts have reported on coagulase-negative staphylococci as common causes of peritonitis, accompanied by increasing methicillin resistance and changing susceptibility among Gram-negative bacilli [30,31]. A nine-year Brazilian analysis demonstrated temporal changes in Gram-negative antibiotic susceptibility, emphasising the risks of maintaining fixed empirical regimens without periodically reviewing local resistance data [31]. Other Brazilian studies have identified fungal peritonitis as an uncommon but severe complication associated with catheter loss and mortality. *Candida parapsilosis* was the leading species, although a wide range of yeasts were also isolated [32].

Mexican reports have described particularly severe outcomes following *E. coli* peritonitis, including poor response to broad-spectrum therapy in some cohorts. A retrospective study involving 2 PD units looked at 49 episodes of *E. coli* peritonitis before and after 1996; it demonstrated increasing virulence leading to poorer outcomes after 1996 [33].

### 2.6. Africa

Evidence from Africa remains comparatively limited and is predominantly derived from single-centre cohorts, largely due to a smaller number of established PD programmes and reduced access to microbiological surveillance. Nevertheless, available data reveal high peritonitis burdens and substantial diagnostic challenges in some settings.

A single centre retrospective South African CAPD (Continuous Ambulatory Peritoneal Dialysis) study reported peritonitis episodes at 1.45 episodes per patient-year, with Gram-positive organisms as the most identified pathogens accounting for 73% of the episodes. It also documented an important burden of Gram-negative infection and antibiotic resistance [34]. Other South African cohorts have demonstrated that socioeconomic deprivation, crowded living conditions, HIV infection and limitations in access to PD support may increase peritonitis risk. In one prospective Durban cohort, HIV-positive patients experienced substantially more peritonitis than HIV-negative patients (1.86 vs. 0.76 episodes/person-years, respectively; hazard ratio [HR], 2.41; 95% confidence interval [CI], 1.69–3.45, *p*  <  0.001), with higher rates of peritonitis and organism-specific patterns were influenced by immune status [35].

North African paediatric data have shown a predominance of Gram-positive organisms, particularly *S. aureus*, but also a substantial contribution from *Klebsiella* and *Pseudomonas*, alongside high culture-negative rates and methicillin resistance. It was noted that exit site infections and tunnelled catheter infections were associated with Gram-positive organisms, while there was a significant association between Gram-negative peritonitis and ureterostomy [36]. A Sudanese multicentre study reported culture negativity in almost three-quarters of cultured episodes, demonstrating the diagnostic limitations that can obscure the underlying microbiological spectrum in resource-constrained programmes [37].

These findings should be interpreted with caution, because small cohorts, inconsistent culture methods and limited susceptibility testing restrict comparisons with high-income registries. They nevertheless demonstrate that microbiological trends derived from Australia, Europe or North America cannot be automatically applied globally due to variations in resources. The microbiological profile of PD peritonitis should therefore be regarded as geographic and programme-specific. International guidance provides an essential common framework but empirical treatment should be informed by regular local assessment of organism-specific incidence, susceptibility patterns, culture-negative rates, relapse, catheter removal and peritonitis-associated mortality. This transition from a rigid framework to local microbiology-guided data represents one of the most important changes in PD-peritonitis management over the past decades.

## 3. Antibiotic Resistance: The New Challenge

Antibiotic resistance has evolved as one of the most consequential challenges in the management of PD-associated peritonitis. Historically, empirical regimens covering both Gram-positive and Gram-negative organisms were effective against most organisms encountered within PD programmes. However, repeated antibiotic exposure, recurrent peritonitis, hospitalisation and the increasing circulation of resistant organisms have reduced the reliability of conventional empirical treatment. The challenge is no longer simply to identify the causative organism but to ensure that the initial regimen remains active against an increasingly heterogeneous resistance profile. The nature of resistance varies considerably between countries and individual PD centres.

Patients receiving PD are vulnerable to antibiotic resistance. Recurrent peritonitis and repeated courses of broad-spectrum antibiotics create repeated opportunities for colonisation and infection by resistant organisms. Exposure to antibiotics could alter skin, gastrointestinal and environmental microbial reservoirs, predisposing to selection of resistant staphylococci, enterococci and Gram-negative bacilli. In a cohort examining multidrug-resistant organism-associated PD peritonitis, these episodes were associated with lower cure rate, higher relapse rate and higher treatment failure than infections caused by susceptible organisms [38].

Longitudinal surveillance indicates that resistance does not evolve uniformly across organisms or regions. A 15-year single-centre South Korean study demonstrated stable resistance patterns across the years [39], whereas surveillance from a German centre over 32 years identified substantial variation in antibiotic susceptibility despite a predominance of Gram-positive organisms [40]. More recent German data again demonstrated that adequate empirical antibiotic treatment depended heavily on centre-specific organism and susceptibility distributions [41]. Collectively, these studies show why historical microbiological profiles cannot be assumed to remain applicable indefinitely; even where the balance between Gram-positive and Gram-negative infection appears stable, resistance within individual species may change over time.

### 3.1. Resistant Gram-Positive Infection

Methicillin resistance among staphylococci remains one of the most common resistance challenges affecting empirical Gram-positive coverage. Coagulase-negative staphylococci continue to present a substantial proportion of touch-contamination-related episodes. Molecular analysis from a Brazilian PD centre demonstrated a high burden of MRSA. Camargo et al. investigated the molecular epidemiology of CNS-causing PD-associated peritonitis at a single Brazilian centre over 18 years (1995–2011), identifying 115 CNS-related episodes among 878 peritonitis episodes, including 72 caused by *Staphylococcus epidermidis*. The mecA gene, which confers methicillin resistance, was detected in 55 bacterial isolates (47.8%), with resistance becoming more common in the later study years [42]. CNS remains a common cause of touch-contamination-related infection, but they increasingly harbour the *mecA* gene and associated staphylococcal cassette chromosome *mec* element. This has important clinical implications, explaining the bacterial genetic significance conferring resistance and virulence.

Vancomycin remains an important therapeutic option for MRSA infection, but increasing reliance on glycopeptides has important stewardship implications. In a cohort of hospitalised PD patients screened during a vancomycin-resistant *Enterococcus* outbreak, 16.9% were colonised with VRE, and vancomycin exposure during the preceding three months was independently associated with VRE colonisation. Although colonisation does not equate to peritonitis, it creates an intestinal reservoir from which invasive infection may subsequently arise and shows the ecological consequences of repeated glycopeptide exposure in PD populations [43].

### 3.2. Resistant Gram-Negative Infection

Resistance among Gram-negative organisms presents an even greater therapeutic challenge because several antibiotics conventionally used for empirical PD-peritonitis treatment may also be compromised. The contemporary concerns are ESBL-producing Enterobacterales, AmpC β-lactamase-producing organisms, carbapenem-resistant Enterobacterales and multidrug-resistant non-fermenting Gram-negative bacilli, particularly *Pseudomonas aeruginosa* and *Acinetobacter* species. Unlike isolated resistance to a single drug, these phenotypes may compromise the effectiveness of cephalosporins, fluoroquinolones and aminoglycosides, substantially narrowing the range of antibiotics that can be administered by the intraperitoneal route.

A single-centre Chinese study evaluating temporal changes in PD-peritonitis microbiology identified important shifts in pathogen distribution and antibiotic susceptibility; although demonstrating decreasing levels of Gram-negative bacilli associated PD peritonitis, it also demonstrated overall reduction in cure rates of these organisms. Antibiotic resistance rates were overall stable [44]. These findings suggest that improvements in general PD practice have not necessarily translated into better outcomes for resistant Gram-negative infection.

The evolution of resistance is particularly well documented in *Escherichia coli*, one of the most frequent causes of Gram-negative PD peritonitis. In a longitudinal cohort of 253 *E. coli* episodes between 2006 and 2018 in Southern China, resistance to cefazolin and ciprofloxacin increased significantly over time, while the proportion of ESBL-producing isolates rose from 34.7% to 46.8%. ESBL-producing isolates demonstrated substantially broader co-resistance than non-ESBL organisms, including resistance to cephalosporins, quinolones and aminoglycosides [45].

Non-fermenting Gram-negative bacilli are particularly difficult to manage. *Pseudomonas aeruginosa* combines intrinsic resistance, inducible β-lactamase production, porin modification and rapid biofilm formation. Resistance may emerge during treatment, meaning that an isolate initially reported as susceptible can subsequently become resistant under the pressure of antibiotic treatment. A 15-year study from two large PD centres in Southern China confirmed that *Pseudomonas* peritonitis was associated with poor cure rates despite generally preserved susceptibility to several first-line agents [46]. This distinction is important: adverse outcomes in *Pseudomonas* infection are driven not only by conventional antibiotic resistance but also by virulence, catheter colonisation, biofilm formation and difficulty achieving durable source control.

The emerging concept of difficult-to-treat resistant *Pseudomonas* further complicates management. Difficult-to-treat resistance describes non-susceptibility to the principal conventional antipseudomonal β-lactams and fluoroquinolones, leaving few reliably active antibiotic options. Newer β-lactam–β-lactamase inhibitor combinations may retain activity against selected isolates, but evidence regarding their intraperitoneal administration, stability in PD solutions and clinical effectiveness in PD peritonitis remains limited. A contemporary review of complicated PD peritonitis emphasised that resistant *Pseudomonas* infection frequently requires early consideration of catheter removal rather than repeated escalation of antibiotic treatment alone [47].

*Acinetobacter* represents another major resistance threat. In a Hong Kong cohort of PD-associated *Acinetobacter* peritonitis, 18% of isolates were multidrug-resistant, and 8% were carbapenem-resistant [12].

UK experience has long demonstrated the importance of laboratory diagnosis of gram-negative peritonitis and the need for centre-specific susceptibility surveillance. In an early British microbiological study of isolates recovered from patients with 32 consecutive CAPD-associated peritonitis episodes in 1988, the authors compared culture techniques and found that centrifuging 50 mL of dialysate followed by leukocyte lysis produced the highest culture yield, with 84% of samples positive; filtration of the same volume without lysis was almost as effective. By contrast, enrichment culture yielded 65% positivity, while conventional centrifugation without leukocyte lysis yielded only 59% positivity after 48 h, probably because organisms became trapped and died within leukocytes. The study also reported that intraperitoneal vancomycin plus ceftazidime was safe and effective, with symptoms usually resolving by day 4, supporting the use of a regimen that covers both Gram-positive and Gram-negative organisms while awaiting culture results [48]. Although predating the modern era of ESBL and carbapenemase dissemination, this study provided some of the earliest UK evidence that Gram-negative susceptibility in PD required direct microbiological surveillance rather than extrapolation from Gram-positive treatment protocols.

## 4. Evolving Pharmacological Management

In the 1970s, one of the major pharmacological developments was the consolidation of the intraperitoneal (IP) route as the preferred method of antibiotic delivery in patients without systemic sepsis. Intraperitoneal administration produces high local concentrations while maintaining systemic exposure and facilitates outpatient treatment [3]. Antibiotics may be administered continuously or intermittently depending on their pharmacokinetic profile, but appropriate dosing requires consideration of dwell time, residual kidney function and PD modality.

**Figure 1:**
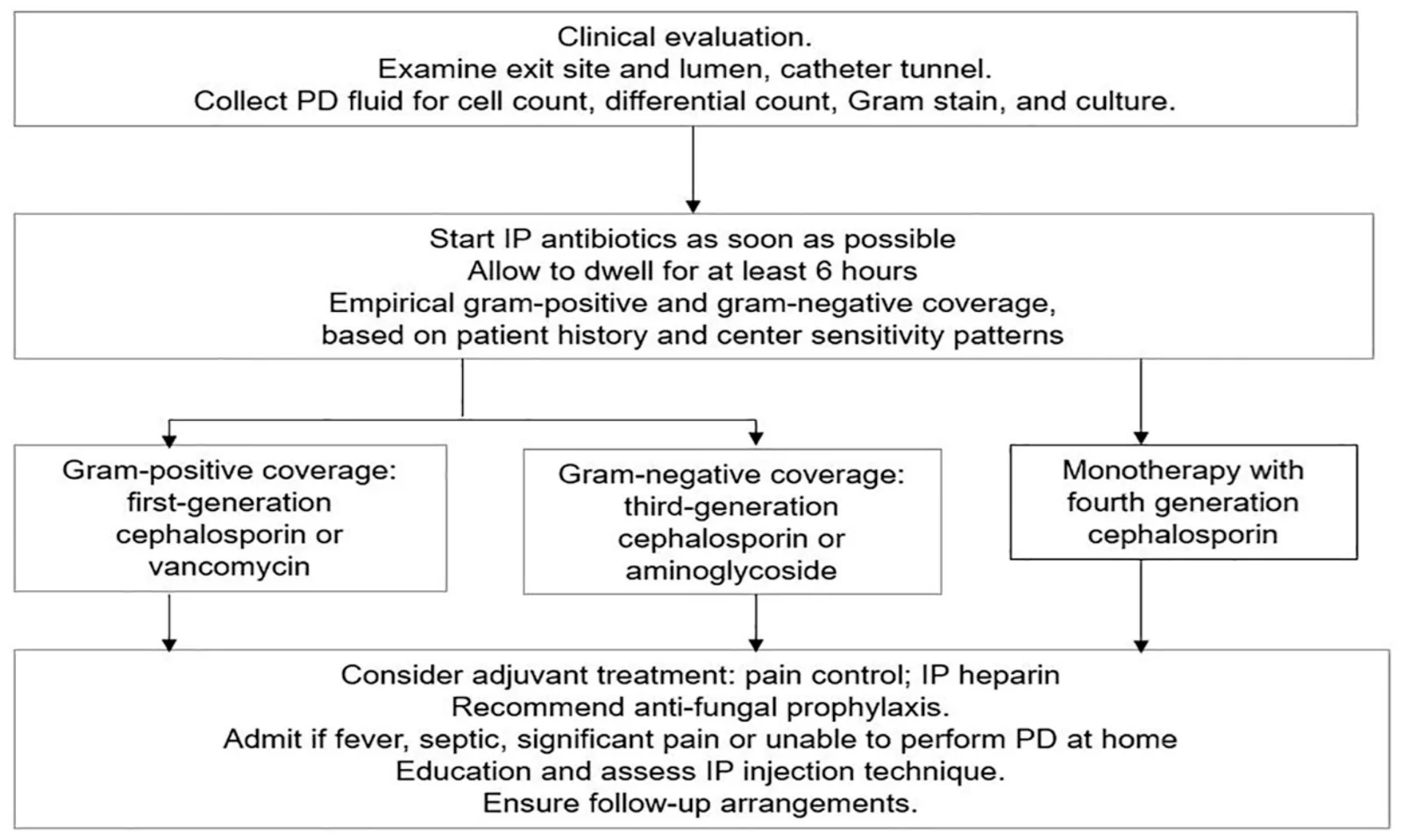
Taken from ISPD 2022 PD peritonitis guidelines: The algorithm of initial management for PD patients presenting with a clinical diagnosis of peritonitis [3]. PD: peritoneal dialysis.

The pharmacological management of PD-associated peritonitis has evolved from relatively fixed empirical antibiotic combinations towards a more individualised strategy incorporating local susceptibility patterns, intraperitoneal pharmacokinetics, organism-specific therapy, therapeutic drug monitoring and antibiotic stewardship. Although immediate empirical coverage of both Gram-positive and Gram-negative organisms remains the cornerstone of treatment allowing it to dwell for a period of 6 h as per ISPD’s 2022 guidelines (Figure 1), contemporary practice increasingly prioritises optimisation of intraperitoneal drug exposure [3].

Historically, empirical therapy commonly combined cephalosporins or vancomycin with either an aminoglycoside or ceftazidime. Randomised trials subsequently demonstrated that several regimens could achieve comparable efficacy. Leung et al. in the early 2000s found similar cure rates with imipenem/cilastatin monotherapy and cefazolin plus ceftazidime, although the ecological consequences of routine carbapenem use limit its role as standard empirical therapy [49]. Similarly, intraperitoneal cefazolin plus netilmicin was comparable with cefazolin plus ceftazidime, with no significant detrimental effect on residual renal function from short-course aminoglycoside exposure [50]. More recently in 2019, a multicentre, open-label, noninferiority randomised controlled trial demonstrated that intraperitoneal cefepime monotherapy was non-inferior to cefazolin plus ceftazidime, showing that simplified single-agent therapy may be effective where local bacterial susceptibility permits [51].

Systematic reviews nevertheless show that no empirical regimen is universally superior. The available trials are generally small and heterogeneous, while treatment success varies substantially according to organism and local resistance patterns. In 2014, one proportional meta-analysis study demonstrated superiority of glycopeptide plus ceftazidime for initial therapy of PD peritonitis but no statistical significance over other combinations of various regimes [48,52]. This has shifted practice away from universal antibiotic combinations towards unit- and programme-specific empirical protocols informed by recent local microbiological surveillance.

Pharmacokinetics of antibiotics has become particularly important with the expansion of automated PD. Short nocturnal dwells may alter drug exposure compared with continuous ambulatory PD, from which many historical dosing recommendations were derived. Pharmacokinetic studies of intraperitoneal cefazolin and ceftazidime have shown that therapeutic concentrations in plasma and dialysate can be achieved during short-dwell exchanges but have emphasised the need to adapt dosing to contemporary dialysis prescriptions rather than extrapolate directly from CAPD [53].

Vancomycin and aminoglycosides illustrate the transition from fixed dosing towards pharmacokinetic optimisation. Vancomycin is commonly administered intermittently because of its prolonged half-life in kidney failure, but substantial interpatient variability in absorption and clearance supports therapeutic drug monitoring. Aminoglycosides remain effective for susceptible Gram-negative organisms, although their use raised some concerns back in the early 2000s, when the ISPD peritonitis guidelines then recommended that aminoglycosides not be used in patients with residual renal function due to cumulative ototoxicity, nephrotoxicity and residual kidney function. Short courses have not consistently been associated with accelerated loss of residual renal function, supporting their continued selective use where local susceptibility favours them [50,54].

Treatment has also become increasingly organism-specific. Once microbiological results are available, unnecessary empirical agents should be discontinued and therapy narrowed. Coagulase-negative staphylococcal peritonitis generally requires shorter treatment, whereas *Staphylococcus aureus*, enterococcal and *Pseudomonas* infections require longer or more specialised regimens. *Pseudomonas* peritonitis usually requires two active agents from different classes, while resistant or biofilm-associated infection may require early catheter removal rather than repeated escalation of antibiotic therapy [3].

Another important change has been recognition of fungal peritonitis as a preventable complication of antibacterial exposure. Antibiotic courses disrupt normal microbial flora and increase the risk of subsequent fungal infection. One of the early randomised trials in 1996 demonstrated that oral nystatin prophylaxis reduced antibiotic-associated Candida peritonitis [55], and more recent meta-analysis supports antifungal prophylaxis during systemic or intraperitoneal antibiotic treatment [56]. Antifungal prophylaxis is consequently now integrated into peritonitis prevention strategies rather than being considered separately from antibacterial treatment [3].

The emergence of multidrug-resistant organisms has further expanded the therapeutic repertoire. Linezolid and daptomycin may be required for resistant Gram-positive infection, while carbapenems, polymyxins and newer β-lactam–β-lactamase inhibitor combinations may be considered for selected resistant Gram-negative organisms [47]. However, PD-specific evidence for newer agents remains limited. Systemic efficacy cannot automatically be extrapolated to PD because treatment requires adequate intraperitoneal penetration, stability in dialysis fluid and compatibility with glucose-, icodextrin- and neutral-pH solutions.

Compatibility and stability of antibiotics in dialysate is increasingly important as newer antibiotic agents are introduced. Reviews of antibiotic stability in PD solutions demonstrate substantial variation according to drug, concentration, PD solution composition, temperature and storage duration [57]. Ling et al. conducted a systematic review of 1254 abstracts, including 28 studies and identifying 18 antibiotic agents, to assess whether less commonly used antibiotics remain chemically, physically, microbiologically, and therapeutically stable when added to peritoneal dialysis solutions. Of the nine antibiotics with intraperitoneal dosing recommendations in the ISPD guidelines, only seven had stability data applicable to practice: cefotaxime, ceftriaxone, daptomycin, ofloxacin, and teicoplanin were stable in glucose-based solutions; tobramycin only in Extraneal; and fosfomycin in Extraneal, Nutrineal, and Physioneal 1.36% and 2.27% solutions. The review concludes that antibiotic stability cannot be assumed across different dialysis fluids, particularly icodextrin-based, low-glucose-degradation-products and pH-neutral solutions; thus, culture-directed intraperitoneal therapy should be guided by drug-specific compatibility data [57,58]. For many newer agents, clinical evidence remains restricted to pharmacokinetic studies, case reports and small case series. The pharmacological challenge is therefore not simply identifying an active antibiotic but ensuring that it can be delivered reliably and safely through the intraperitoneal route [58].

UK studies have contributed particularly to understanding aminoglycoside exposure and therapeutic drug monitoring. As previously described, Baker et al. found that empirical aminoglycoside treatment for PD peritonitis was not associated with accelerated loss of residual renal function, supporting short-course use when clinically appropriate [54]. Blunden et al. subsequently demonstrated some variability in vancomycin and gentamicin concentrations despite guideline-based dosing, with both subtherapeutic vancomycin and elevated gentamicin levels occurring in routine practice [59]. This provided an important UK rationale for therapeutic drug monitoring and individualised dosing.

The London Peritonitis Audit further demonstrated that treatment outcomes varied markedly according to organism and antibiotic strategy, with particularly poor cure rates for *Pseudomonas* infection and better outcomes in centres using intraperitoneal rather than predominantly oral regimens [21]. Although these studies predate the current multidrug-resistance landscape, they helped establish principles that remain central to modern management: adequate intraperitoneal exposure, organism-directed treatment and avoidance of empirically ineffective therapy.

Over the past years, pharmacological management of PD peritonitis has therefore evolved from broadly standardised empirical combinations towards a more individualised model integrating local microbiology, intraperitoneal pharmacokinetics, therapeutic drug monitoring, organism-specific treatment, antifungal prophylaxis and antibiotic stewardship. Future progress will depend less on simply broadening antibiotic coverage and more on generating robust PD-specific pharmacokinetic and clinical evidence for contemporary and emerging therapies.

The development of antibiotics active against resistant organisms has outpaced their evolution in PD. Agents including linezolid, daptomycin, tigecycline, ceftaroline, ceftolozane–tazobactam and ceftazidime–avibactam provide potential options against selected resistant pathogens, but most have not been evaluated in adequately powered PD-peritonitis trials. Evidence is frequently extrapolated from other systemic infections. A dedicated review of newer antibiotics for drug-resistant PD peritonitis found that the supporting evidence largely consisted of individual cases, small case series, in vitro compatibility studies and pharmacokinetic reports [60].

A Cochrane review demonstrated results from systematic reviews of randomised controlled trials (RCTs) and quasi-RCTs, which could not identify any optimal antibiotic treatment, but glycopeptide regimens were more likely to achieve a complete cure than a first-generation cephalosporin [61].

## 5. Practical Take-Home Messages

Several practical considerations arise from the evolving evidence on PD-associated peritonitis. First, the general principles of peritonitis treatment are similar for patients receiving CAPD and APD, but the pharmacokinetics of antibiotics may differ. Short APD dwell times could reduce intraperitoneal antibiotic exposure; therefore, adequate dwell time, appropriate dosing and, where indicated, supplemental dosing should be considered rather than directly extrapolating CAPD regimens to APD. In patients receiving rapid-cycle APD, the proportion of polymorphonuclear leukocytes in dialysate may also be more diagnostically informative than the absolute white-cell count [3].

Second, culture-negative peritonitis should not initially be assumed to represent a non-infectious or “sterile” process. Appropriate cultures should be obtained before antibiotic treatment whenever possible, and persistent culture negativity should encourage reassessment of sampling methodology and investigation for fastidious bacteria, fungi and mycobacteria. Where microbiological investigation remains negative and the clinical presentation is atypical, non-infectious causes of cloudy effluent, including eosinophilic or chemical peritonitis, haemoperitoneum and other intra-abdominal inflammatory processes, should be considered [3].

Third, the identification of an atypical organism in an exit-site or tunnel infection does not itself mandate routine catheter replacement. Management should reflect the causative organism, antibiotic susceptibility, clinical response and the presence of concomitant peritonitis. The 2023 ISPD catheter-related infection recommendations advise catheter removal when an exit-site or tunnel infection progresses to, or occurs simultaneously with, peritonitis caused by the same organism. For catheter-related infection that fails to resolve with effective antibiotic therapy, simultaneous catheter removal and reinsertion may be considered, while surgical salvage strategies, such as cuff removal/shaving with a new exit site, may be appropriate in selected patients [62]. Atypical infections, such as non-tuberculous mycobacteria, require extra caution, as they may need prolonged multidrug antimicrobial therapy and catheter removal [62].

Finally, fungal peritonitis should be regarded differently from most bacterial episodes because effective source control is essential. Once fungi are identified in PD effluent, prompt PD catheter removal and appropriate antifungal treatment are recommended, usually necessitating temporary transfer to haemodialysis [3]. Nevertheless, fungal peritonitis should not automatically be regarded as permanent PD cessation; following complete resolution of infection, carefully selected patients may subsequently undergo catheter reinsertion and return to PD.

## 6. Conclusions

Over the years, PD-associated peritonitis incidence has declined overall, but important regional differences in microbiology, antibiotic resistance and treatment outcomes persist. Gram-positive organisms remain common, while Gram-negative and fungal infections continue to cause disproportionate PD failure and mortality. Future priorities should focus on stronger antibiotic stewardship, routine surveillance of local resistance patterns, along with more consistent prevention and treatment protocols. Further work is also needed to reduce global centre-level variation and develop targeted strategies for high-risk organisms and recurrent infection.

## Figures and Tables

**Table 1:** Adapted from the PDOPPS, worldwide distribution of organism and class-specific PD peritonitis rates episodes per patient-year.

Country	Australia/NZ	Canada	Japan	Thailand	UK	US
No. of peritonitis episodes	187	393	345	258	120	969
Time at risk, person-years	534.8	1376.8	1257.1	639.6	314.1	3754.6
Peritonitis rate/person-years (95% CI)	0.35 (0.30–0.40) ^a^	0.29 (0.26–0.32) ^b^	0.27 (0.25–0.30)	0.40 (0.36–0.46) ^a^	0.38 (0.32–0.46) ^a^	0.26 (0.24–0.27)
Organisms						
Gram-positive	0.14 (39%)	0.13 (45%)	0.10 (37%)	0.10 (26%)	0.14 (38%)	0.10 (37%)
Gram-negative	0.07 (20%)	0.05 (16%)	0.04 (13%)	0.12 (29%)	0.07 (19%)	0.03 (13%)
Culture-negative	0.05 (14%)	0.04 (16%)	0.06 (21%)	0.11 (28%)	0.05 (14%)	0.04 (16%)
Polymicrobial	0.04 (10%)	0.03 (9%)	0.02 (8%)	0.02 (4%)	0.03 (8%)	0.01 (5%)
Yeast	0.01 (3%)	0.01 (2%)	<0.01 (1%)	0.01 (2%)	0.01 (3%)	<0.01 (1%)
Other	0.05 (14%)	0.03 (11%)	0.05 (19%)	0.05 (12%)	0.07 (19%)	0.07 (28%)
Coagulase-negative *Staphylococcus*	0.052 (15%)	0.045 (16%)	0.014 (5%)	0.023 (6%)	0.048 (13%)	0.054 (21%)
*Staphylococcus aureus*	0.034 (10%)	0.034 (12%)	0.025 (9%)	0.027 (7%)	0.054 (14%)	0.017 (7%)
*Pseudomonas aeruginosa*	0.009 (3%)	0.007 (3%)	0.009 (3%)	0.009 (2%)	0.022 (6%)	0.004 (2%)

Note: Unadjusted rates and 95% CIs based on total event counts and person-years at risk in each country. Values in parentheses represent percent of episodes within country, unless stated otherwise. Abbreviations: CI, confidence interval; NZ, New Zealand; UK, United Kingdom; US, United States. ^a^ *p* < 0.05 and ^b^ *p* < 0.10 versus United States after Dunnett adjustment for multiple comparisons against a common reference group.

## Data Availability

No new data were created or analyzed in this study. Data sharing is not applicable to this article.
